# Right hepatic artery pseudoaneurysm caused by stone extraction–related trauma during endoscopic retrograde cholangiopancreatography: a case report

**DOI:** 10.3389/fmed.2025.1676454

**Published:** 2025-10-29

**Authors:** Wenjie Ou, Xu Han, Ruyi Tan, Yan Huang, Xiang Xiao

**Affiliations:** ^1^Department of Endoscopy Center, The Affiliated Xiangtan County People's Hospital of Changsha Medical University, Xiangtan, Hunan, China; ^2^Department of Gastroenterology II, Jilin Provincial People's Hospital, Changchun, Jilin, China

**Keywords:** endoscopic retrograde cholangiopancreatography, pseudoaneurysm, right hepatic artery, biliary hemorrhage, digital subtraction angiography

## Abstract

Hepatic artery pseudoaneurysm (HAP) is a rare but potentially fatal complication of endoscopic retrograde cholangiopancreatography (ERCP), often misdiagnosed due to its nonspecific presentation. We report a case of a 72-year-old male with choledocholithiasis who developed progressive biliary hemorrhage following ERCP. Initial conservative treatment and placement of a fully covered metal biliary stent failed to control the bleeding. The patient subsequently presented with hemorrhagic shock and significant hemoglobin decline. Computed tomography angiography (CTA) and digital subtraction angiography (DSA) revealed a ruptured right HAP, which was successfully managed by transcatheter arterial embolization (TAE). Detailed retrospective analysis of imaging findings and intraoperative procedures indicated that the HAP likely resulted from iatrogenic vascular injury to the right hepatic artery adjacent to the remnant cystic duct, caused by mechanical trauma during stone retrieval maneuvers. This case highlights the need for early recognition of vascular injury as a source of post-ERCP bleeding, particularly when bleeding persists despite standard endoscopic management. Timely use of CTA and DSA is essential for accurate diagnosis, and TAE offers a safe and effective treatment option.

## Introduction

Endoscopic retrograde cholangiopancreatography (ERCP) has become an internationally recognized therapeutic modality for managing biliary and pancreatic diseases (1, 2). Reported complications including pancreatitis, cholangitis, gastrointestinal tract injuries such as perforation, and hemorrhage (3). The overall incidence of post-ERCP bleeding ranges from 0.3 to 2% (1, 4), with most cases attributed to mucosal bleeding following endoscopic sphincterotomy (EST). Such bleeding is usually manageable through endoscopic interventions such as hemostatic drug injection, electrocoagulation, balloon tamponade, or clip placement. However, arterial bleeding—particularly due to rupture of a hepatic artery pseudoaneurysm (HAP)—is exceedingly rare and often presents as acute, massive hemorrhage that can be life-threatening. The right hepatic artery is anatomically adjacent to the common bile duct and cystic duct, rendering it susceptible to injury during ERCP procedures, particularly during guidewire manipulation, balloon dilation, stone retrieval, or stent deployment.

Right HAP secondary to ERCP is extremely rare, with only a few cases documented in the literature. This article presents a case of right hepatic artery pseudoaneurysm rupture and hemorrhage following ERCP. We review the clinical course, explore the possible pathogenesis in conjunction with a literature review, and discuss diagnostic and therapeutic strategies, aiming to enhance clinician awareness of this rare but potentially fatal complication.

## Case information

**Figure 1:**
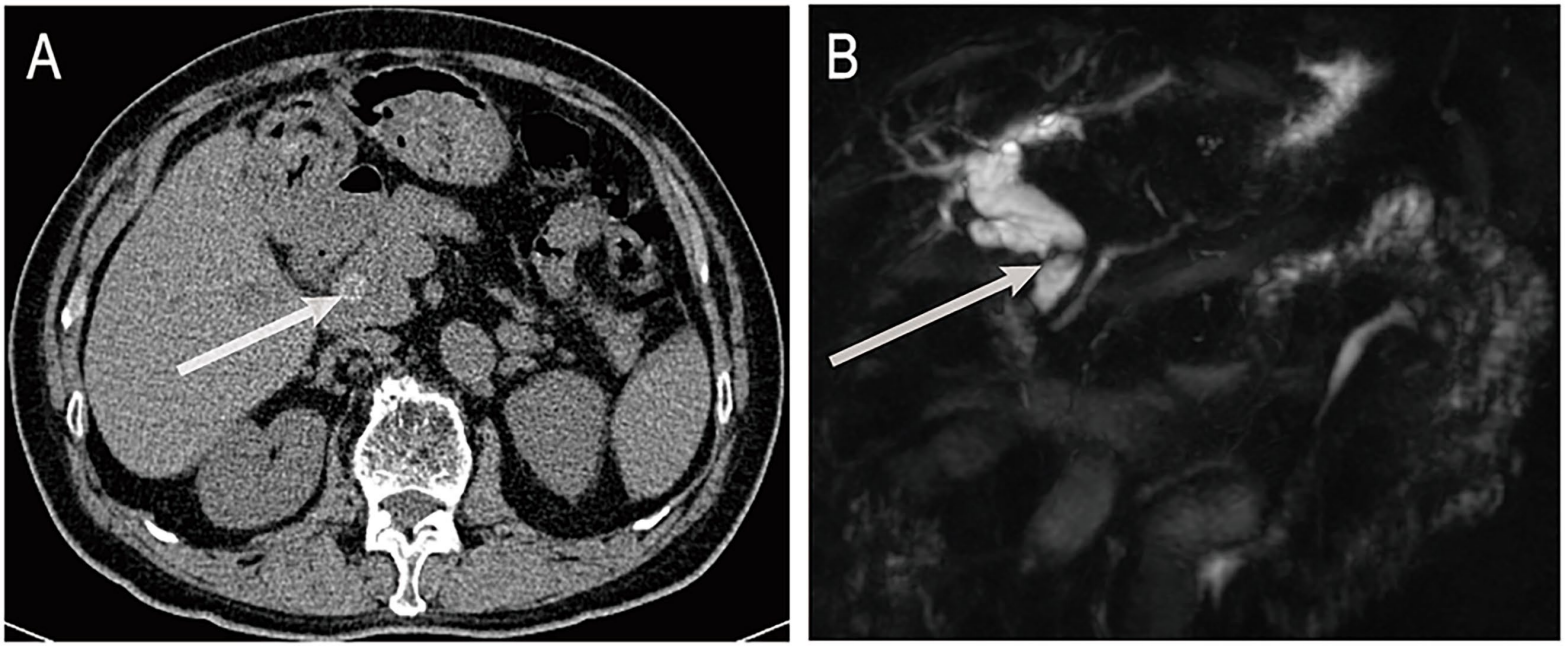
**(A)** Abdominal non-contrast CT shows a stone in the distal common bile duct. **(B)** MRCP reveals dilatation of intrahepatic and extrahepatic bile ducts with distal obstruction caused by a stone. ERCP, endoscopic retrograde cholangiopancreatography; CT, computed tomography; MRCP, magnetic resonance cholangiopancreatography. The white arrows indicate bile duct stones.

A 72-year-old male was admitted on June 16, 2025, with a 3-day history of fever. His medical history included cholecystectomy, previous ERCP, and chronic obstructive pulmonary disease. On admission, his body temperature was 38.8 °C, accompanied by cough without significant sputum production or abdominal pain. Laboratory tests revealed a neutrophil ratio of 92.1% and an absolute neutrophil count of 8.49 × 10^9^/L. Blood cultures were positive for *Escherichia coli*. Liver function tests showed a total bilirubin level of 45.9 μmol/L, direct bilirubin of 33.1 μmol/L, and indirect bilirubin of 12.8 μmol/L. Aspartate aminotransferase was elevated to 259 U/L and alanine aminotransferase to 161 U/L. Abdominal computed tomography (CT) revealed choledocholithiasis and biliary obstruction (Figure 1A). Magnetic resonance cholangiopancreatography (MRCP), including non-contrast and contrast-enhanced sequences, demonstrated intra- and extrahepatic bile duct dilation, common bile duct stones, and reduced enhancement of the left hepatic lobe, consistent with biliary infection (Figure 1B).

**Figure 2:**
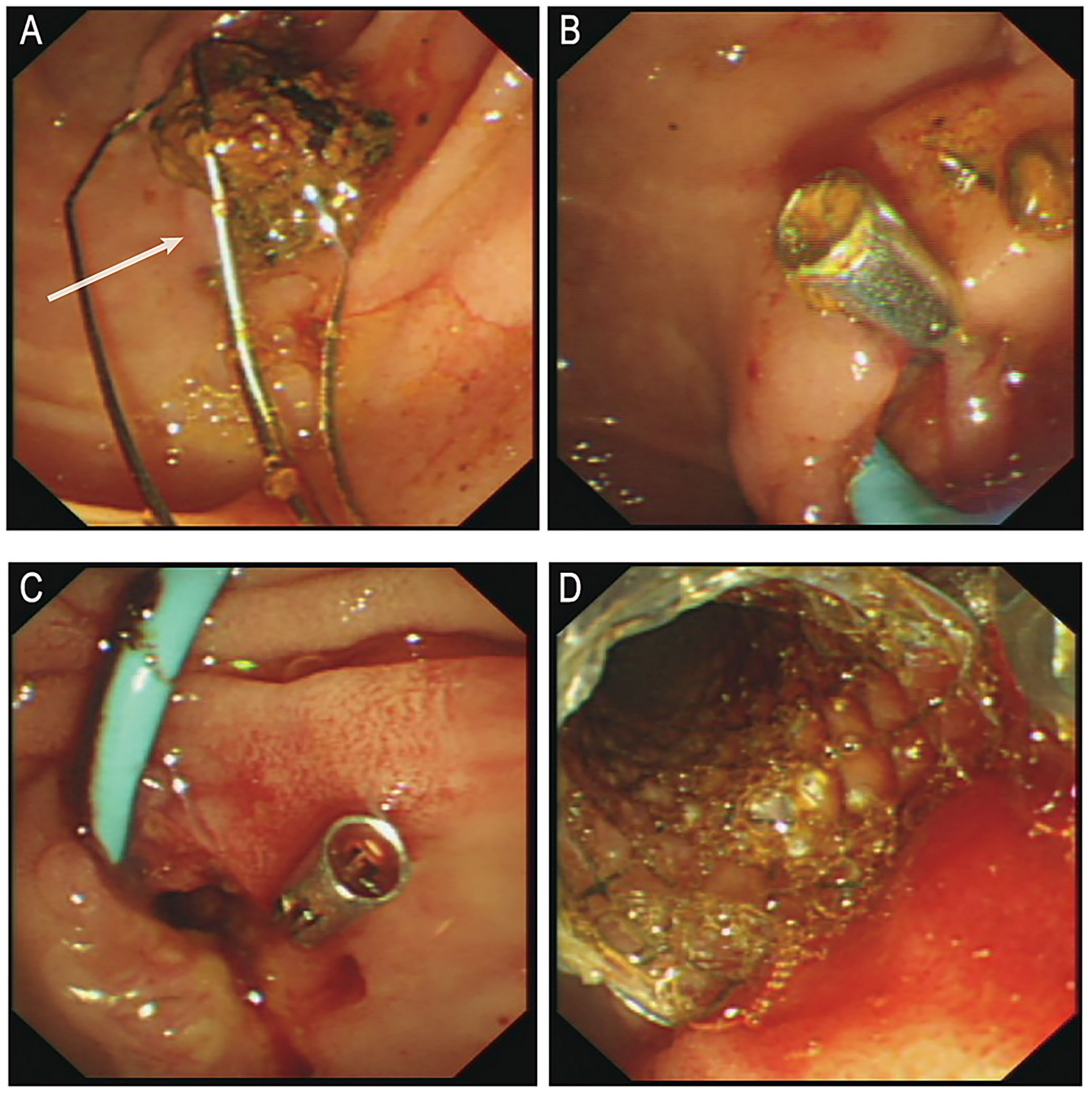
**(A)** Post-stone extraction. **(B)** Post-operative placement of titanium clamps and nasal bile duct drainage tube. **(C)** Active bleeding observed in the bile duct. **(D)** Post-hemostasis following metal stent placement. ERCP, endoscopic retrograde cholangiopancreatography. White arrows indicate the extracted stone.

Following anti-infective and supportive treatment, ERCP was performed on June 25. During the procedure, endoscopic sphincterotomy was carried out, and a cylindrical balloon with a diameter of 1.1 cm was used to dilate both the distal common bile duct and the duodenal papilla. A stone retrieval basket (Four-line spiral type) was then employed to successfully extract a common bile duct stone measuring approximately 1.5 × 1.6 cm (Figure 2A). After the procedure, one titanium clip was applied at the sphincterotomy site to prevent post-EST bleeding, and a nasobiliary drainage tube was placed for bile drainage (Figure 2B). Six hours after the first ERCP, the patient’s serum amylase and lipase levels were transiently elevated (240 U/L [normal range: 35–135 U/L] and 455 U/L [normal range: 1–60 U/L], respectively), but both values returned to normal within 10 h (109 U/L and 62 U/L, respectively).

On postoperative day 1, a small amount of bloody bile was observed through the nasobiliary drainage tube, and hemoglobin (Hb) decreased to 120 g/L. This was initially attributed to biliary mucosal erosion, and the patient was kept under observation. In the afternoon, approximately 100 mL of bleeding occurred. Epinephrine combined with hemostatic agents was administered via nasobiliary lavage, along with intravenous hemostatic therapy. However, later that evening, the patient experienced two episodes of melena, characterized by black stools mixed with dark red clots, totaling approximately 500 g, as well as one episode of hematemesis (approximately 50 mL of dark red gastric contents). The nasobiliary tube continued to drain dark, blood-tinged bile. Hb dropped further to 93 g/L, and subsequently to 77 g/L. Emergency transfusion of 2 units of packed red blood cells was administered. At 00:20 the following day, an emergency ERCP was performed under local anesthesia. Intraoperatively, profuse active bleeding and intraductal thrombi were observed (Figure 2C). After irrigation, a 6 cm fully covered metal biliary stent was placed over the guidewire (Figure 2D). Postoperatively, no further bleeding was observed, and the patient was transferred to the intensive care unit for close monitoring.

**Figure 3:**
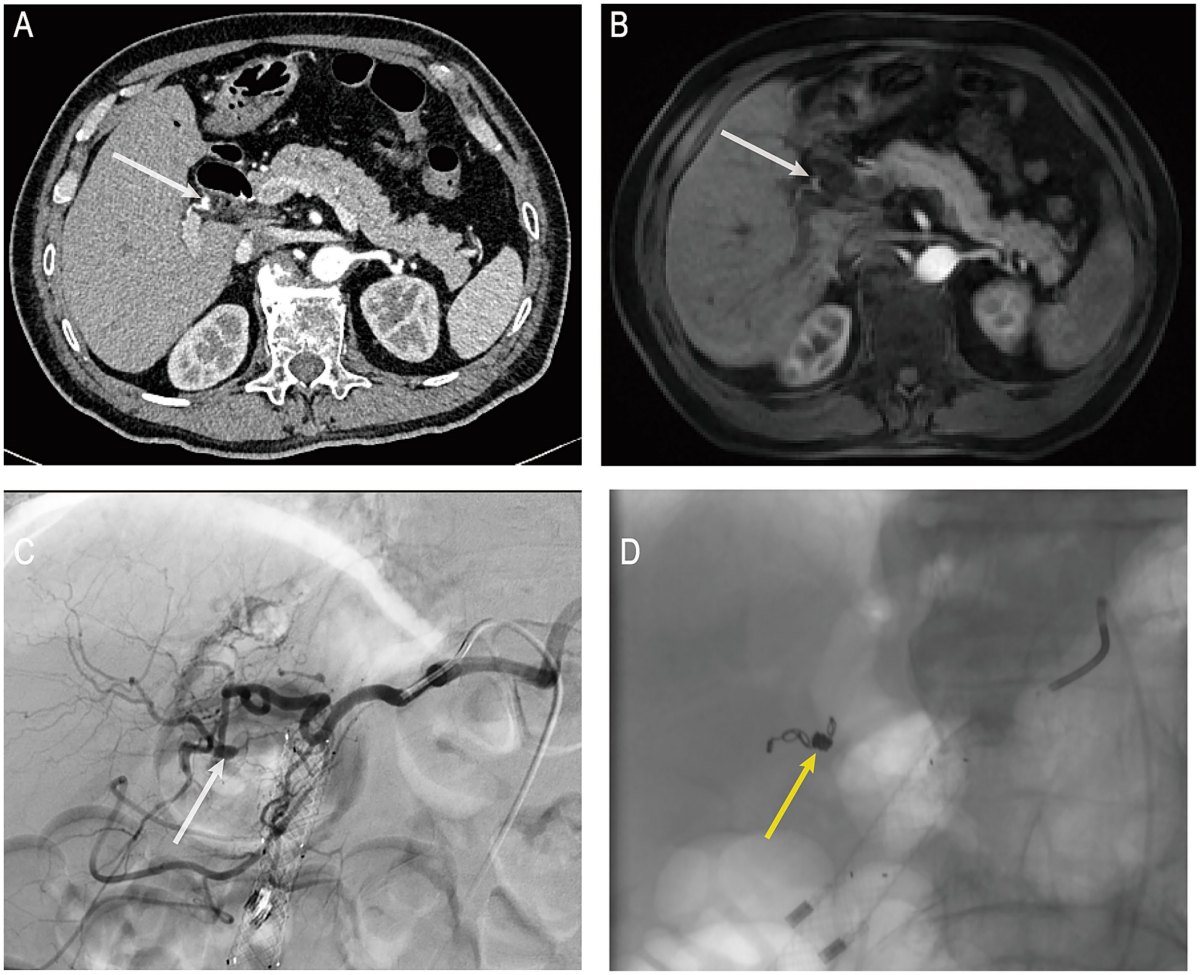
**(A)** Post-hemorrhage hepatic artery CTA. **(B)** Pre-hemorrhage enhanced MRCP. **(C)** Post-hemorrhage hepatic artery DSA. **(D)** Post-TAE under DSA guidance. CTA, computed tomography angiography; MRCP, magnetic resonance cholangiopancreatography; DSA, digital subtraction angiography; TAE, transcatheter arterial embolization. White arrows indicate the location of the pseudoaneurysm in the right hepatic artery; yellow arrows indicate the site of arterial embolization.

On the day of the procedure, the patient’s blood pressure dropped to 84/53 mmHg, accompanied by altered consciousness, clammy skin, and other signs of hemorrhagic shock. Hb further declined to 73 g/L, indicating ongoing active biliary bleeding. Emergency computed tomography angiography (CTA) revealed a mildly dilated, nodular change in the proximal right hepatic artery measuring approximately 4 × 4 mm (Figure 3A), suggestive of an aneurysm or HAP. No aneurysm had been identified on pre-ERCP MRCP imaging (Figure 3B), supporting the likelihood that the lesion developed secondary to ERCP-related procedural trauma. Following a multidisciplinary consultation, emergency digital subtraction angiography (DSA)–guided hepatic artery angiography and transcatheter arterial embolization (TAE) were performed under local anesthesia. DSA demonstrated a pseudoaneurysmal outpouching of a branch of the right hepatic artery, approximately 3 mm in diameter (Figure 3C). After superselective catheterization with a microcatheter, a 2–4 × 40 mm coil was deployed distally, followed by the placement of six 2–3 × 20 mm coils within the aneurysmal sac. Proximal embolization was further reinforced with the injection of gelatin sponge particles. Post-embolization angiography showed no residual aneurysmal filling (Figure 3D). Postoperatively, the patient’s vital signs stabilized, he was transferred back to the general ward, Hb rose to 92 g/L, abdominal pain subsided, and liver function gradually improved. On postoperative day 10 after TAE, the biliary metal stent was endoscopically removed without evidence of rebleeding.

## Discussion

Visceral artery aneurysms are rare but potentially fatal vascular lesions, with hepatic artery aneurysms accounting for approximately 20% of such cases (5). These can be further classified into true aneurysms and HAP. With the increasing prevalence of laparoscopic and hepatobiliary interventional procedures, the proportion of HAPs caused by blunt or penetrating trauma has significantly risen in recent years (5, 6). As a routine screening modality, CTA offers valuable insights into the morphology and size of the aneurysm, as well as its spatial relationship with adjacent structures—facilitating early detection of HAP. In contrast, DSA is widely regarded as the gold standard for both diagnosis and treatment, enabling simultaneous localization and endovascular embolization (7, 8).

The pathogenesis of HAPs is diverse, with contributing factors including prior laparoscopic cholecystectomy (9), pancreatitis (10), tumors (11), and, as highlighted in this case, direct vascular trauma from guidewire or stone retrieval devices during ERCP (12, 13). Haseeb et al. (12) described a left hepatic segment-2 branch pseudoaneurysm presenting as delayed post-ERCP hemobilia, successfully managed with coil embolization. They proposed both procedural trauma and inflammation-related vascular fragility as possible causes and advised avoiding deep guide-wire advancement to reduce vascular injury risk. Li et al. (13) reported a left hepatic artery pseudoaneurysm identified on day 6 after ERCP, with the authors attributing the injury to guide-wire penetration of the biliary tree. In contrast, Zhao et al. (14) summarized three HAPs unrelated to ERCP (two after laparoscopic cholecystectomy and one after trauma), underscoring the heterogeneity of peribiliary vascular injury. All these factors may disrupt the arterial wall, leading to pseudoaneurysm formation under intraluminal pressure. Due to the rarity of HAP, the initial post-ERCP finding of bloody bile through the nasobiliary drain in this patient was misattributed to biliary mucosal erosion. Conservative medical treatment was initially adopted without early vascular imaging. However, persistent hemorrhage necessitated emergency ERCP, during which hemostatic clips and a 6 cm fully covered metal biliary stent were placed at commonly affected bleeding sites—the papilla and the mid-to-distal bile duct. Several studies have demonstrated the effectiveness of covered metal stents in controlling hemorrhage from these sites (2, 15). Despite this intervention, the patient’s bleeding persisted, accompanied by progressive hemoglobin decline, melena, hematemesis, and hypotension consistent with hemorrhagic shock. Following multidisciplinary consultation, CTA was performed and revealed a ruptured HAP in the right hepatic artery. TAE is currently considered the first-line treatment for HAP due to its minimal invasiveness, accurate localization, and rapid hemostatic effect (14). In this case, after the diagnosis was confirmed by DSA, the pseudoaneurysm was successfully treated via superselective embolization using microcoils combined with gelatin sponge particles, resulting in effective hemostasis and favorable clinical recovery.

In this case, the patient experienced persistent hemorrhage following two ERCP procedures, which was ultimately confirmed by DSA to be due to rupture of a right HAP—an extremely rare but critical condition. Multidisciplinary discussion and retrospective imaging analysis revealed no apparent abnormality in the relevant artery before ERCP. However, a new pseudoaneurysm measuring approximately 3 mm in diameter appeared postoperatively, strongly suggesting a causal relationship with intraoperative ERCP manipulation. The patient had a history of cholecystectomy, and imaging demonstrated that the right hepatic artery pseudoaneurysm was located adjacent to the remnant cystic duct (Supplementary material S1). Combined with an analysis of the procedural steps, it was considered that the stone retrieval basket may have inadvertently entered the remnant cystic duct during the procedure. The repetitive advancement and withdrawal of the device likely produced indirect transmission of mechanical stress through the bile duct wall, rendering the adjacent right hepatic artery susceptible to shear and traction forces. These biomechanical stresses, compounded by local inflammatory edema and vascular fragility induced by pre-existing cholangitis, probably caused focal arterial wall disruption and subsequent pseudoaneurysm formation. Such HAPs, when ruptured, typically present as hemobilia rather than intraperitoneal hemorrhage, and are easily misinterpreted as gastrointestinal bleeding or oozing from the EST site. Accurate diagnosis relies on CTA or DSA. Therefore, in cases of unexplained biliary hemorrhage following ERCP, especially when accompanied by progressive hemoglobin decline, clinicians should maintain a high index of suspicion for HAP. Early CTA or DSA should be performed to confirm the diagnosis and enable timely intervention, which is essential for saving the patient’s life.

## Data Availability

The original contributions presented in the study are included in the article/Supplementary material, further inquiries can be directed to the corresponding author.

## Supplementary material

Supplementary Table 2 Actual image of the rock-taking net basket.docx.

Supplementary Video 1 ERCP Stone Removal Intervention Video.mp4.

Supplementary Video 2 ERCP Stone Removal Intervention Video.mp4.

Supplementary Video 3 ERCP Stone Removal Intervention Video.mp4.

Supplementary Video 4 Video of Nasobiliary Drain Placement.mp4.

Supplementary Image 1 Anterior View of the Duodenal Papilla.jpg.
