# Supplementary figures and images for: Right hepatic artery pseudoaneurysm caused by stone extraction–related trauma during endoscopic retrograde cholangiopancreatography: a case report
Source: Front Med (Lausanne). 2025 Oct 29;12:1676454. doi: 10.3389/fmed.2025.1676454 (PMC12605407; doi:10.3389/fmed.2025.1676454)

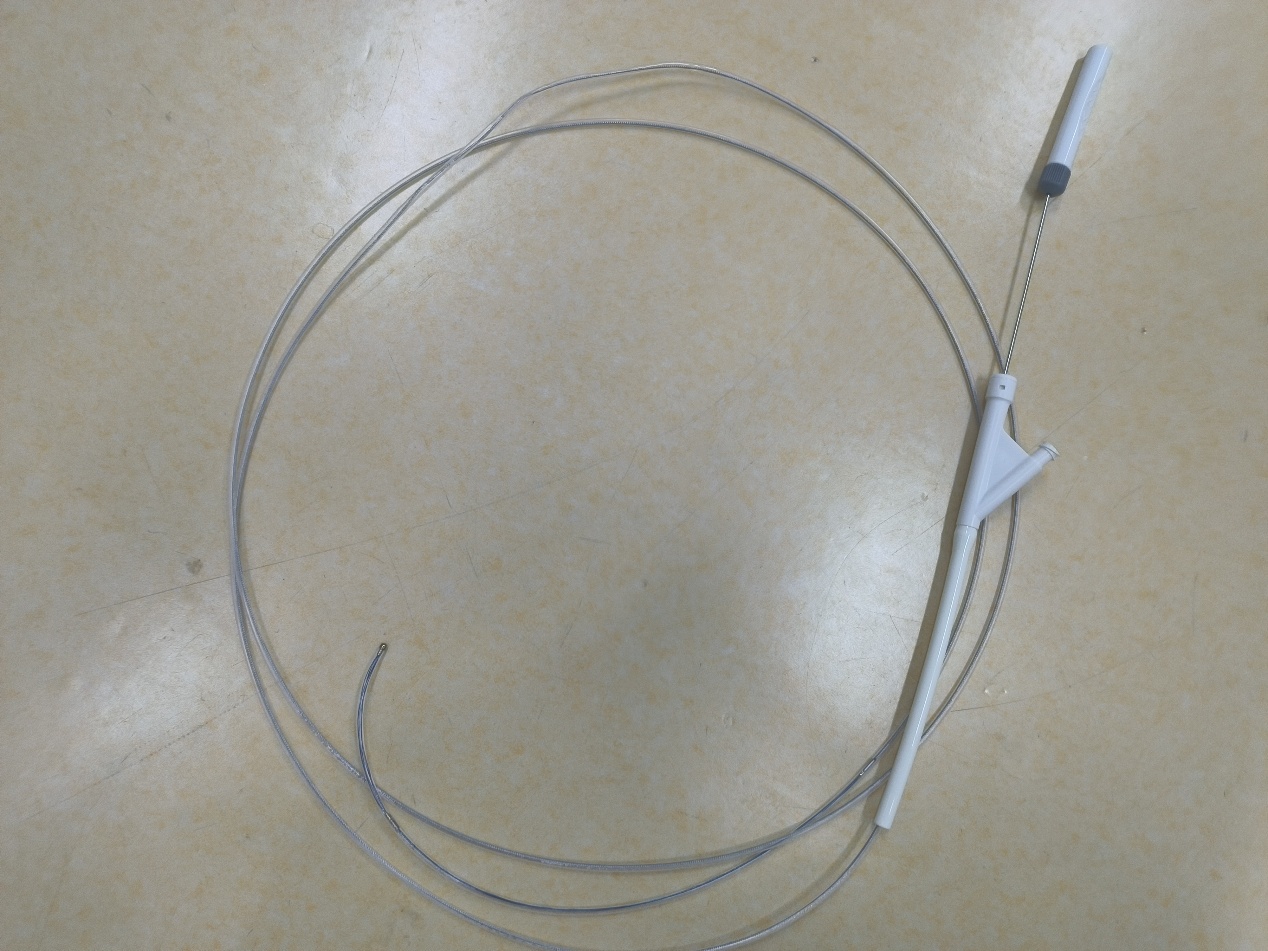

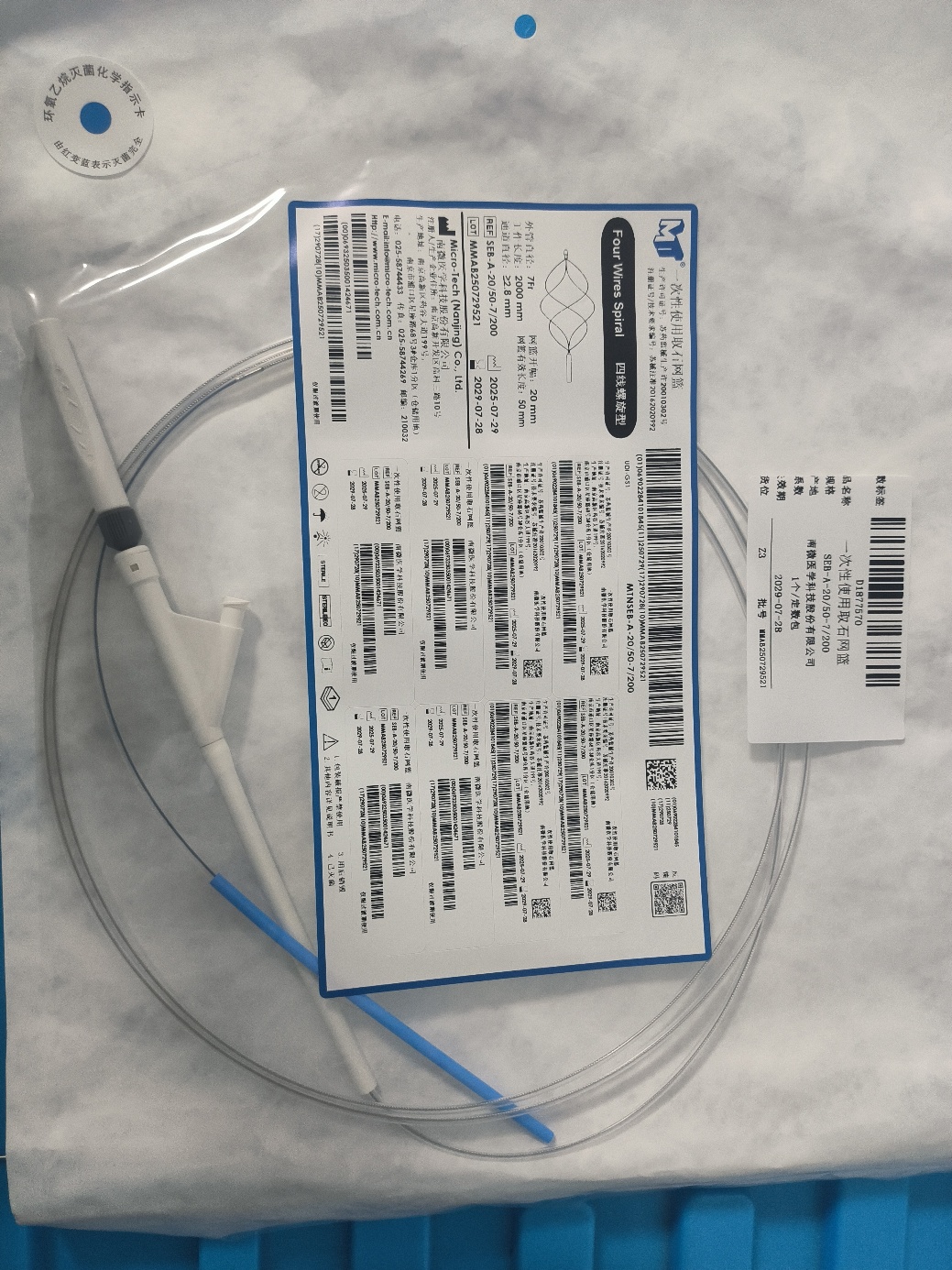


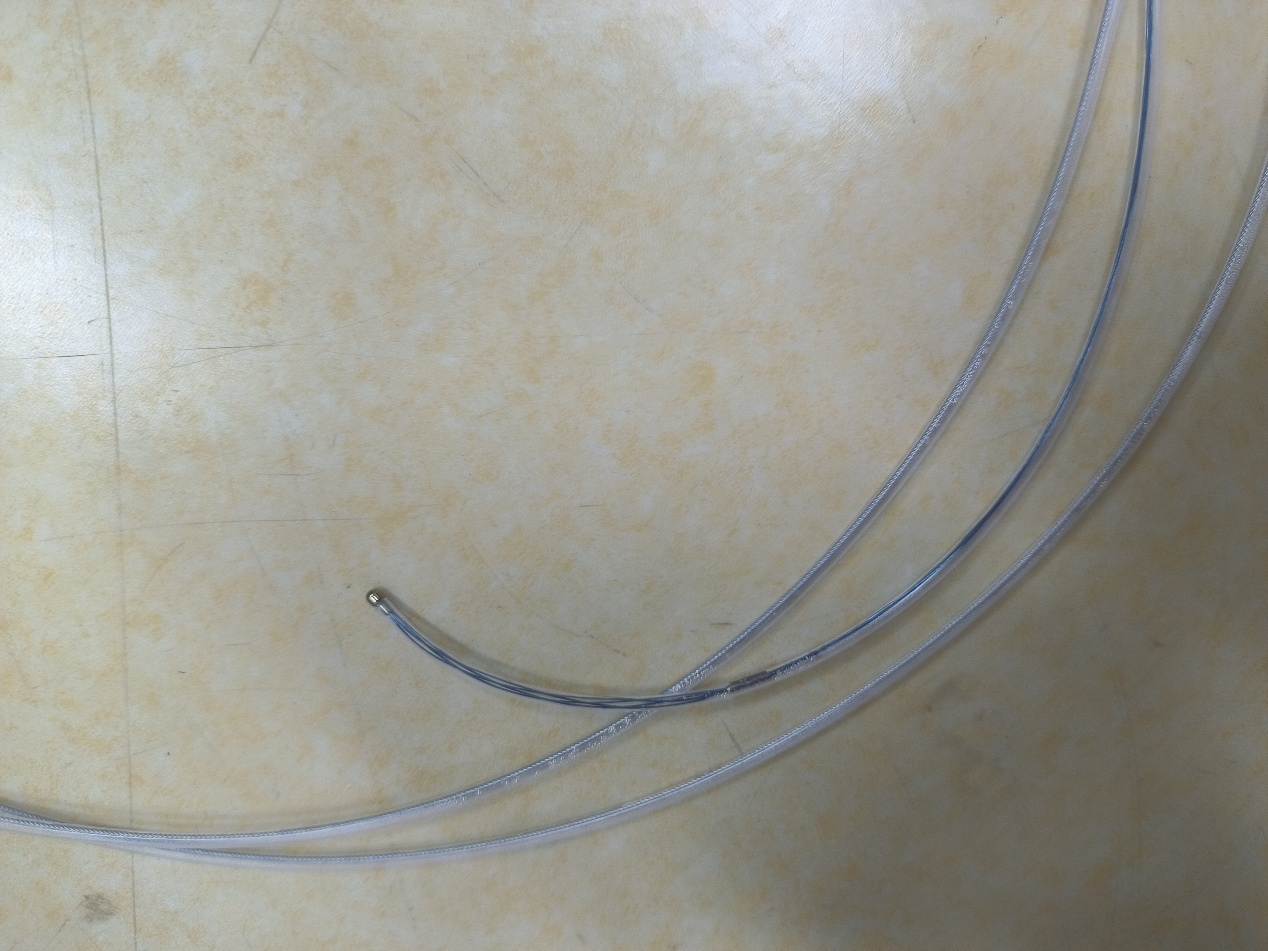

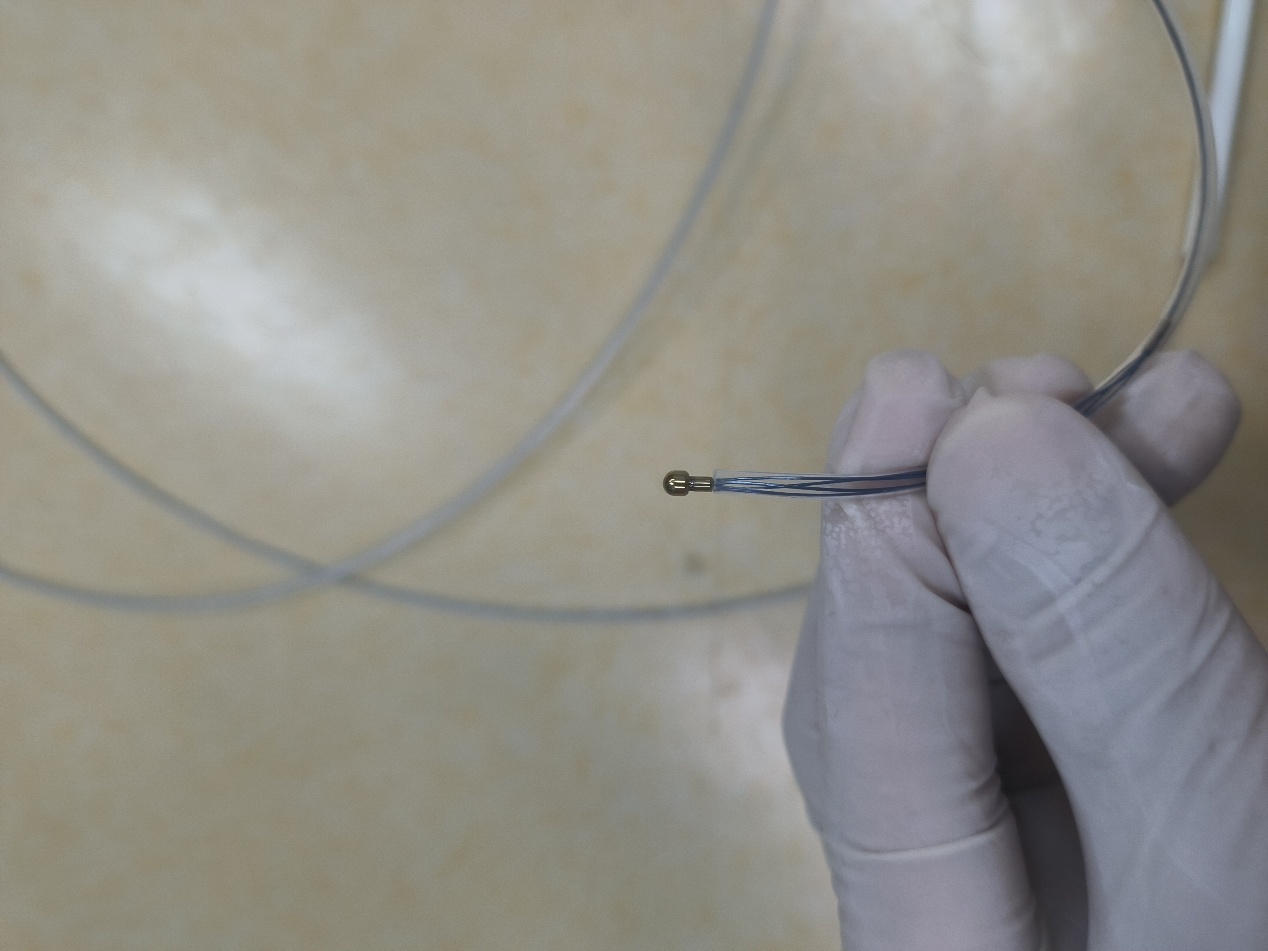


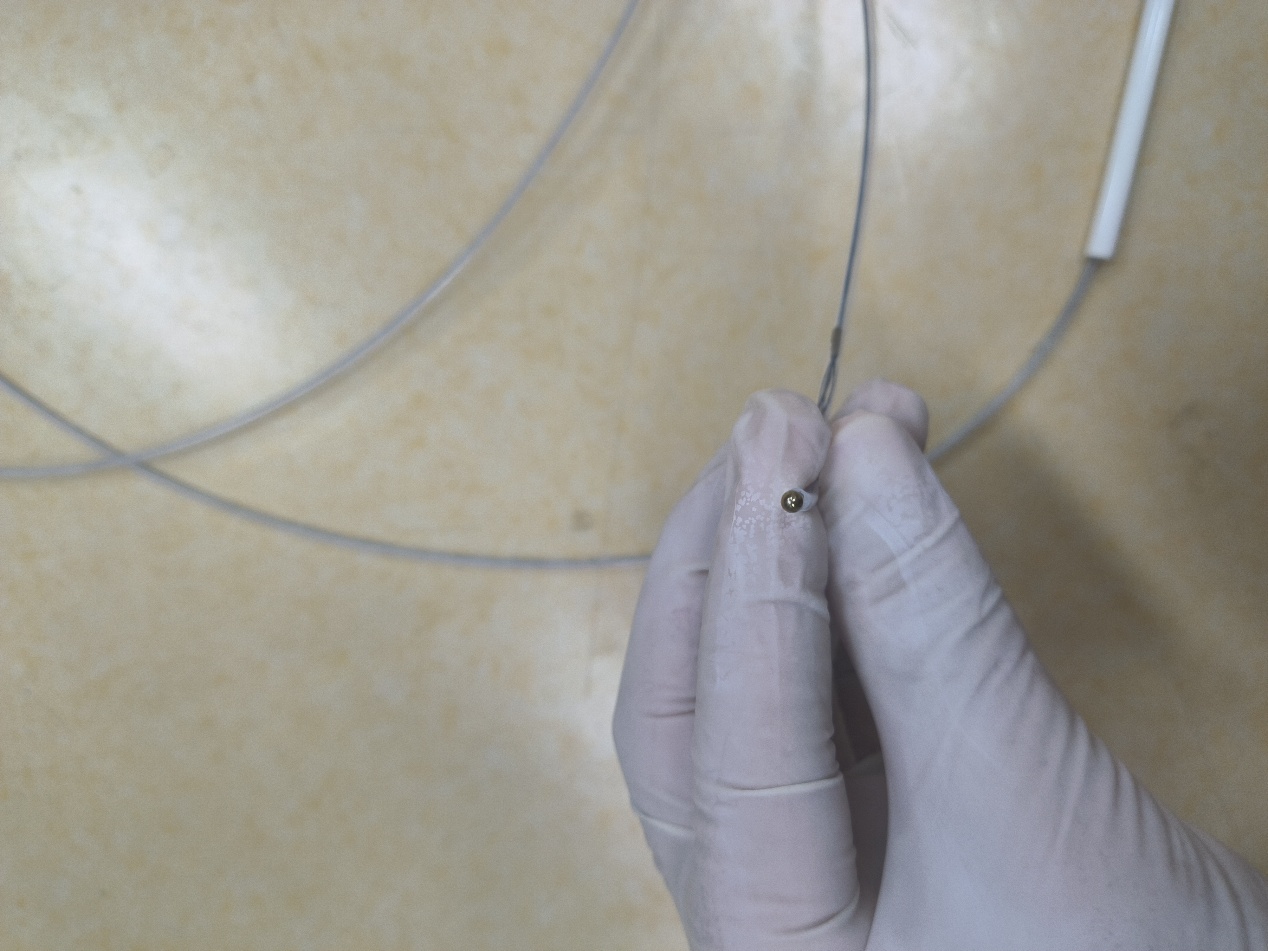


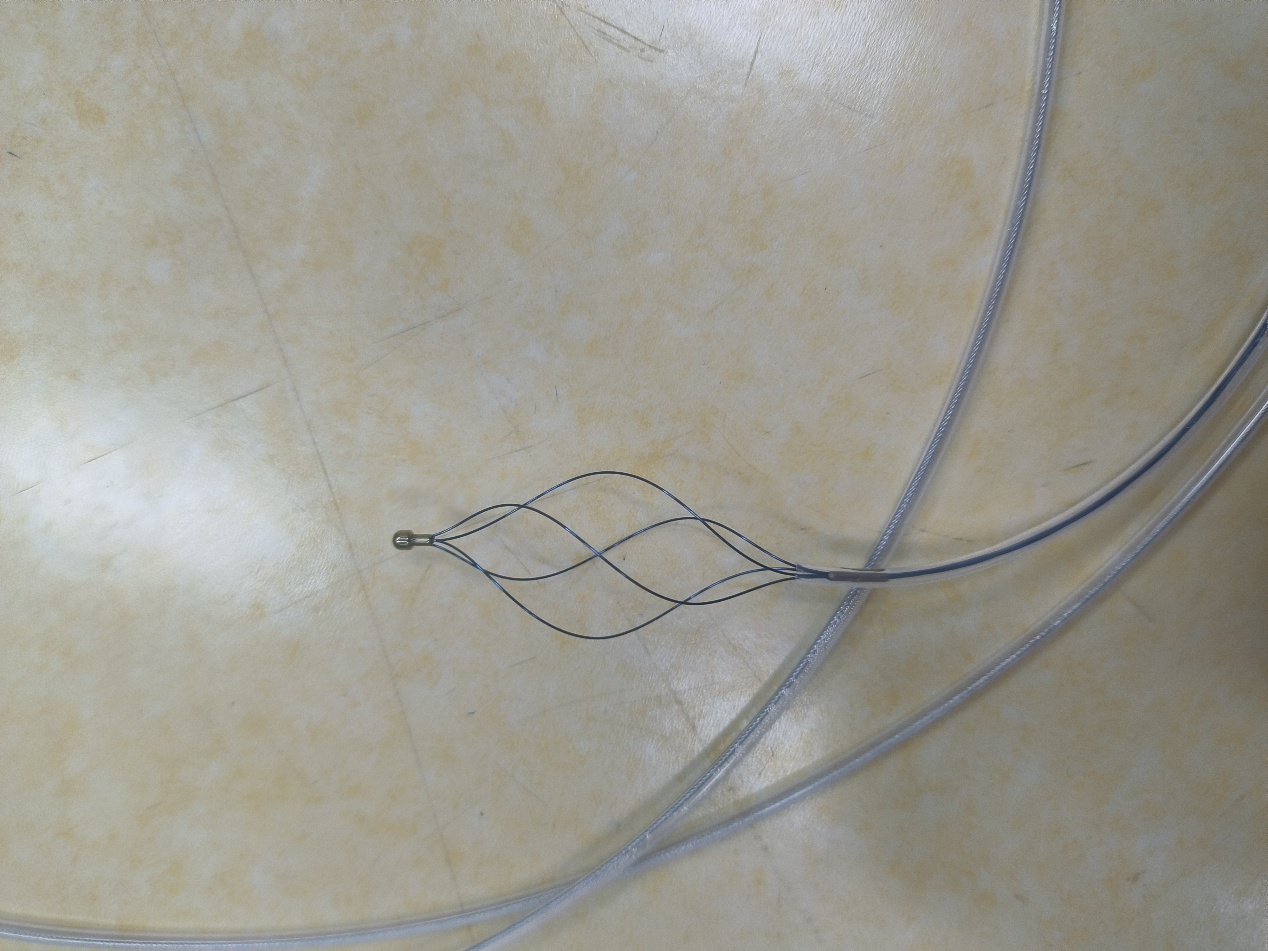


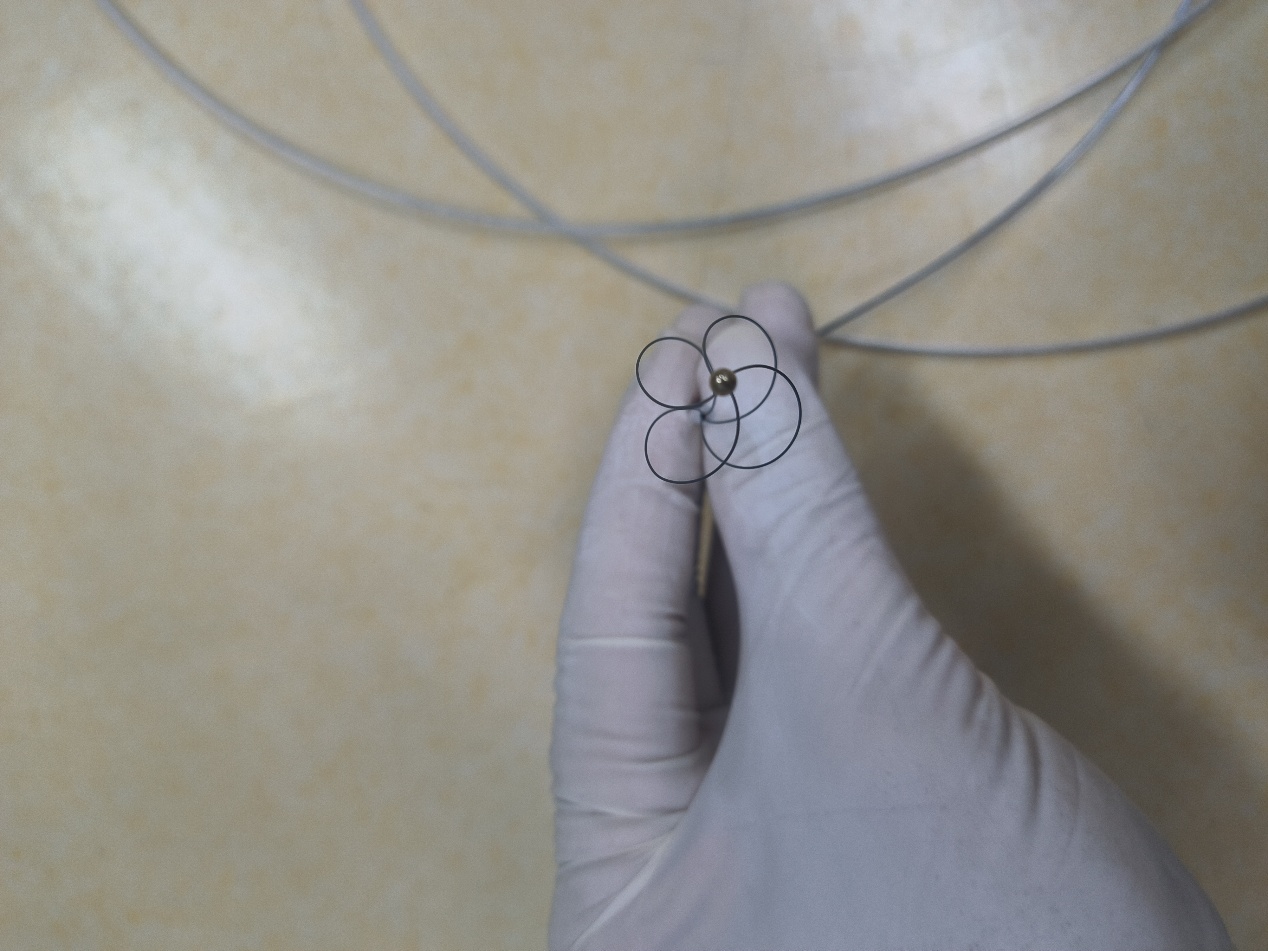

Supplement: Supplementary Table 2 — Actual image of the rock-taking net basket.docx. [file Table_2.DOCX]

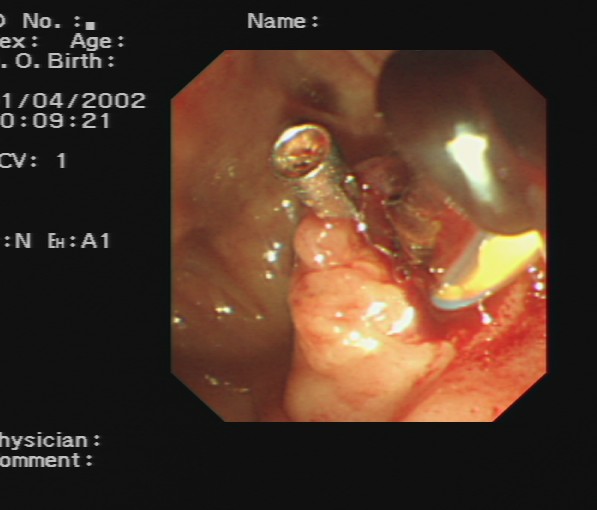

Supplement: Supplementary Image 1 — Anterior View of the Duodenal Papilla.jpg. [file Image_1.JPEG]
