# Supplementary material for: Right hepatic artery pseudoaneurysm caused by stone extraction–related trauma during endoscopic retrograde cholangiopancreatography: a case report
Source: Front Med (Lausanne). 2025 Oct 29;12:1676454. doi: 10.3389/fmed.2025.1676454 (PMC12605407; doi:10.3389/fmed.2025.1676454)
Supplement: Supplementary file 7 [file Table_1.DOCX]

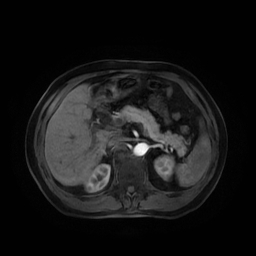

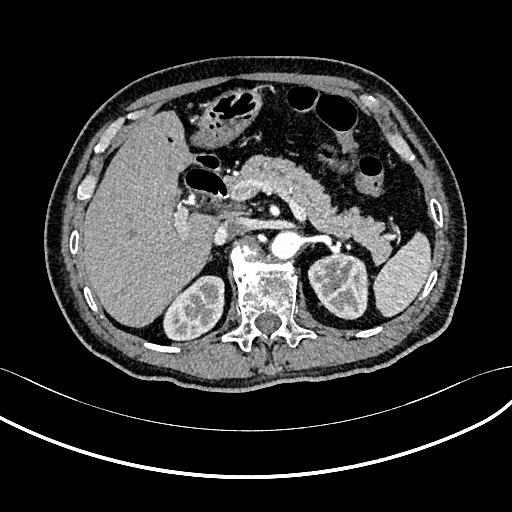
 Figure 1. Axial MRCP image before pseudoaneurysm formation of the right hepatic artery (left) and axial CTA image after pseudoaneurysm formation (right), indicated by white arrows.


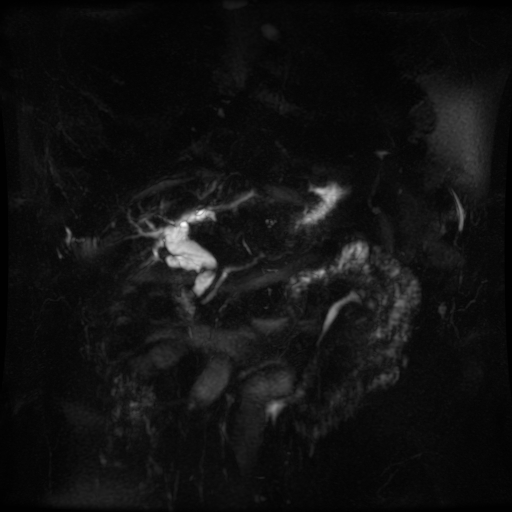
 Figure 2. MRCP image of the entire bile duct shows the presence of the cystic duct stump, indicated by the white arrow.


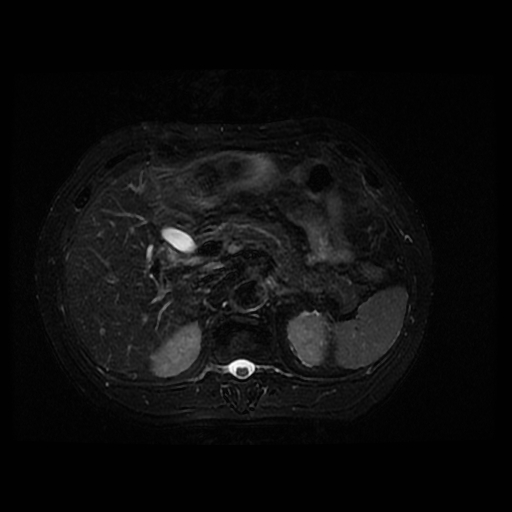
 Figure 3. Axial MRCP image. The white arrow denotes the visualized bile duct. Comparison with Figure 1 confirms this level corresponds to the HAP location in the right hepatic artery based on surrounding anatomical landmarks (pancreas, kidneys, spleen, liver).

Figure 4. This layer is the next layer below Figure 3, where the gallbladder duct stump is visible, indicated by white arrows.
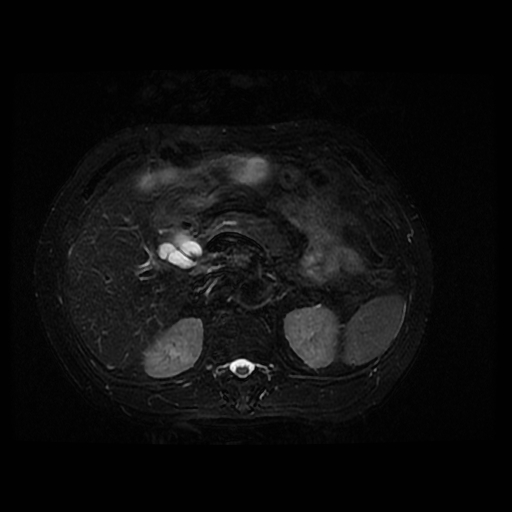


Based on comprehensive medical records and imaging studies, no pseudoaneurysm was identified preoperatively. A pseudoaneurysm occurs when a tear in the blood vessel wall, caused by trauma or other factors, forms a hematoma around the artery. This hematoma is encapsulated by the outer layer of the vessel wall, creating a bulging structure resembling an aneurysm. This pseudoaneurysm developed following endoscopic retrograde cholangiopancreatography (ERCP) stone extraction. Observation of Figures 3 and 4 reveals that the cystic duct stump is immediately adjacent to the pseudoaneurysm below. This anatomical proximity suggests that during the ERCP procedure, the stone retrieval basket may have inadvertently entered the residual cystic duct. Subsequent rapid advancement and withdrawal maneuvers likely caused blunt or penetrating injury to the adjacent right hepatic artery, subsequently leading to pseudoaneurysm formation.
